# HSP60 possesses a GTPase activity and mediates protein folding with HSP10

**DOI:** 10.1038/s41598-017-17167-7

**Published:** 2017-12-05

**Authors:** Tomoya Okamoto, Hiroshi Yamamoto, Ikuru Kudo, Kazuya Matsumoto, Masafumi Odaka, Ewa Grave, Hideaki Itoh

**Affiliations:** 1 Department of Life Science, Graduate School and Faculty of Engineering Science, Akita University, Akita, 010-8502 Japan; 2 Department of Applied Chemistry, Graduate School and Faculty of Engineering Science, Akita University, Akita, 010-8502 Japan

## Abstract

The mammalian molecular chaperone, HSP60, plays an essential role in protein homeostasis through mediating protein folding and assembly. The structure and ATP-dependent function of HSP60 has been well established in recent studies. After ATP, GTP is the major cellular nucleotide. In this paper, we have investigated the role of GTP in the activity of HSP60. It was found that HSP60 has different properties with respect to allostery, complex formation and protein folding activity depending on the nucleoside triphosphate present. The presence of GTP slightly affected the ATPase activity of HSP60 during protein folding. These results provide clues as to the functional mechanism of the HSP60-HSP10 complex.

## Introduction

Molecular chaperones play essential roles in protein homeostasis^1,2^. The protein folding activity of GroEL and its co-chaperone, GroES, have been well characterized both structurally and functionally^3–5^. The tetradecameric barrel-shaped GroEL, which is composed of two heptameric rings stacked back-to-back, binds to GroES in an ATP-dependent manner and generally forms an asymmetric bullet-type complex. Based on protein substrates that hardly fold, it has been reported that GroEL/ES forms a symmetric football-type complex, but the folding cycle of this complex is still controversial^6–8^.

Mammalian HSP60, the eukaryotic homolog of the bacterial chaperonin GroEL, is mainly located in mitochondria and is involved in many intra- and extra-cellular activities^9–14^. It has been reported that mammalian HSP60, in contrast to GroEL, mainly exists as single heptameric ring^15^. X-ray crystallography demonstrated that HSP60 is converted to a tetradecameric double-ring structure in the presence of ATP, and HSP60 forms a football-type complex when both ATP and the co-chaperone, HSP10, are present^16–18^. Taken together, these studies strongly indicate that the ATP-dependent functional cycle of the HSP60/HSP10 complex is quite different from that of the bacterial GroEL/ES complex^19^.

A kinetic analysis of the GroEL ATPase reaction reported that GroEL has a nested cooperativity composed of both intra-ring positive cooperativity and inter-ring negative cooperativity^20^. Several reports about the mammalian HSP60 suggested that HSP60 and GroEL showed different cooperativity properties^16,19^. We present here studies of the cooperative kinetics of wild-type porcine HSP60 purified from cytosol.

In the intact cell, the concentration of GTP is second only to ATP. In a previous study, we reported that the eukaryotic group II chaperonin TRiC/CCT has GTPase activity and this activity leads to a conformational change^21^. Moreover, it has been reported that the 90 kDa heat shock protein (HSP90) has GTPase activity^22^. We now report that the eukaryotic group I chaperonin HSP60 possesses GTPase activity and this reaction is regulated differently from the ATPase-dependent reaction. In comparison to the ATPase activity that produces the stable double-ring structure of HSP60 with HSP10, along with productive refolding of the denatured substrate proteins^16–18^, the GTPase activity of HSP60 was structurally and functionally quite different. These results demonstrate the importance of the ATPase-dependent cycle of HSP60 in protein folding and a supporting function for the GTPase activity.

## Results

### ATP and GTP show different allosteric properties with HSP60

**Figure 1:**
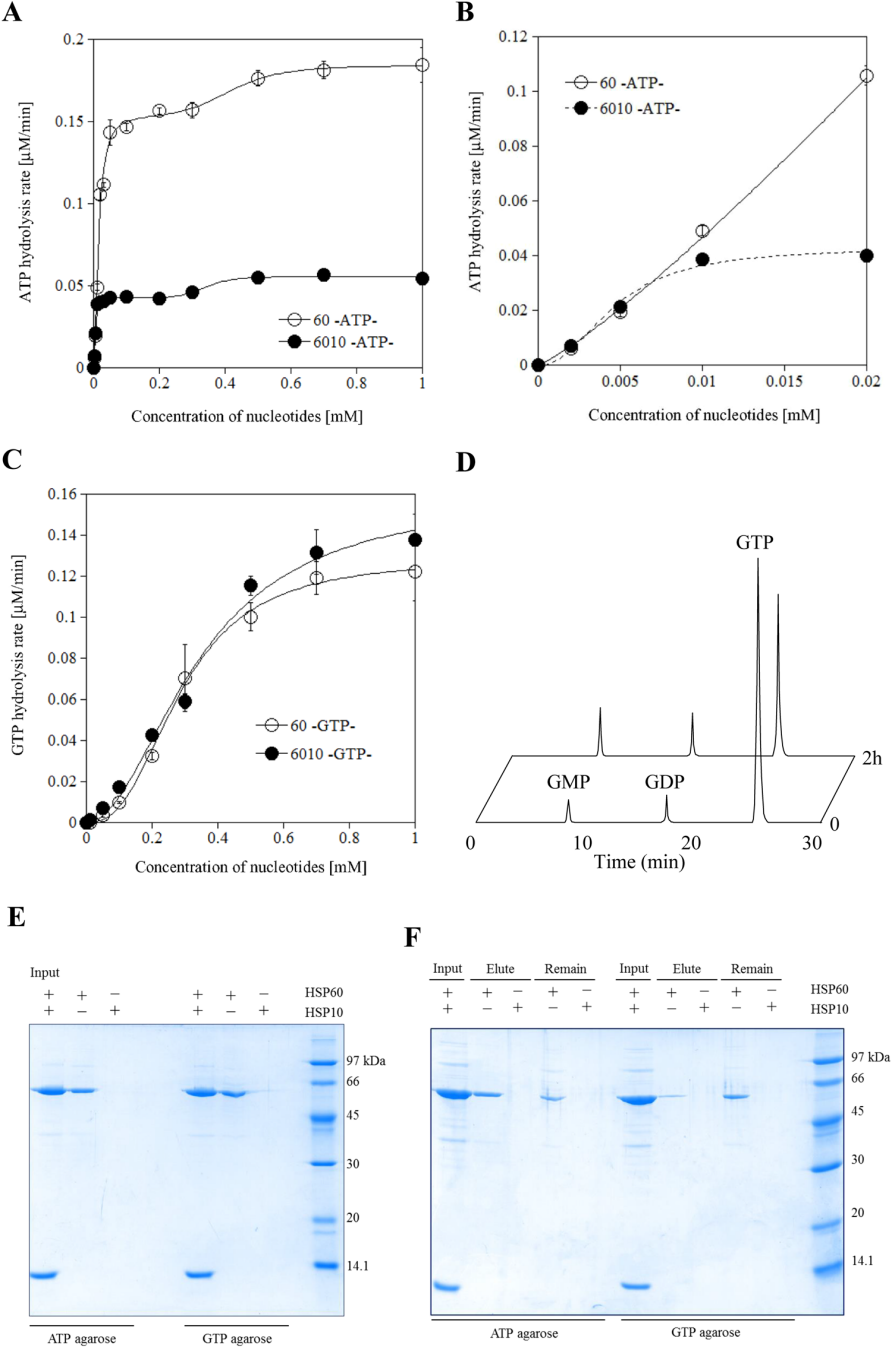
GTPase activity of HSP60 and physiological functions. (**A**), ATPase activity of HSP60 in the presence or absence of HSP10. The kinetic parameters were calculated by curve fitting to the Hill equation 1 or 2 in the Materials and Methods section by Kaleida Graph. (**B**), ATPase activity of HSP60 in the absence or presence of HSP10 in the low concentration range from 0 to 0.02 mM. The kinetic curves were directly fitted to the Hill equation 2. (**C**), GTPase activity of HSP60 in the presence or absence of HSP10 was calculated by curve fitting to the Hill equation 2. (**D**), Purified HSP60 was incubated with GTP at 37 °C for 2 h. Sample of time 0 and 2 h were separated by a C_18_-reverse phase column and absorbance at 256 nm was recorded. (**E**), NTP-pull down assay was performed using ATP- or GTP-agarose in the presence of HSP60 or HSP10. In the current study, to stabilize the interaction between HSP60 and NTP-agarose, a pull down assay was performed in the presence of 0.2 mM AlCl_3_ and 10 mM NaF. Samples were separated by 12% SDS-PAGE and detected by Coomassie Brilliant Blue R250-staining. (**F**), ATP- or GTP-agarose was eluted with 1 mM ATP or GTP in the presence of 0.2 mM AlCl_3_ and 10 mM NaF, respectively.

The nucleotide hydrolysis activity of the bacterial group I chaperonin GroEL and the cytoplasmic group II chaperonin TRiC/CCT have been well investigated^20,23,24^. In the ATP concentration range from 0 to 1.0 mM, the ATPase activity of mammalian HSP60 fit well (Fig. 1A) to a Hill equation for two sequential allosteric transitions (Equation 1 in the materials and methods section), as did TRiC/CCT. Since we and others^16,18^ found that HSP60 mainly exists in the heptameric single ring structure (Figure S1A), we postulated that the first transition observed in ATPase activity corresponds to an intra-ring allosteric transition of single-ring HSP60. The oligomeric equilibrium of HSP60 was shifted from a single to double ring structure in the presence of ATP^18^. The second allosteric transition may reflect the observed ATP-dependent double ring formation. The equilibrium constant of first allosteric transition was calculated to be 14 ( ± 1) μM and that of the second 0.42 ( ± 0.08) mM. The Hill coefficient of the first allosteric transition was calculated to be 1.76 ( ± 0.18) and that of the second allosteric transition was 5.52 ( ± 4.34).

In the case of GroEL, it has been reported that the ATPase activity decreased and the cooperativity in the ATPase of GroEL increased^23^. Inhibition of HSP60 ATPase activity by the co-chaperone HSP10 has been previously reported^18^, and it was also detected in this experiment (Fig. 1A,B and S1B). The ATPase activity of the HSP60/HSP10 complex at ATP concentrations from 0 to 1.0 mM also fit well to Eqn. 1 with an equilibrium constant for the first transition 4.6 ( ± 0.2) μM and the second 0.34 ( ± 0.04) mM. The Hill coefficients were 2.23 ( ± 0.28) and 8.22 ( ± 7.76) for the first and second transitions, respectively. These results were consistent with increased positive cooperativity with respect to ATP of HSP60 in the presence of HSP10, as found previously with GroEL/GroES^23^.

We have previously reported that the cytoplasmic chaperonin TRiC/CCT has GTPase activity and that the structure of CCT is changed in a GTPase-dependent manner as well as the ATPase activity^21^. Figure 1C,D show the GTPase activity of HSP60 in the absence or presence of HSP10. The GTPase activity of this group I chaperonin was also confirmed with the bacterial chaperonin GroEL and the mammalian chaperonin HSP60 (Figure S1C). Different from the result with the ATPase, the GTPase activity of HSP60 fit well the Hill equation (Eqn. 2 in the materials and methods section) and two apparent allosteric transitions as with the ATPase activity were not observed. Moreover, the GTPase activity of HSP60 was not suppressed by HSP10, quite different from the ATPase activity. The GTPase activity of HSP60 formed a clear sigmoidal curve in the absence or presence of HSP10 with Hill coefficients of 2.58 ( ± 0.22) and 1.99 ( ± 0.33), respectively, indicating strong positive cooperativity with respect to GTP. The ATPase activity of HSP60 was suppressed by GDP (Figure S1D), compared to little effect of ADP. Thus, it appears that HSP60 binds to GDP (or GTP) with a low affinity compared to ADP or ATP and that HSP60 has different allosteric behavior with ATP and GTP.

To determine that HSP60 can directly hydrolyze GTP with no contamination, we next investigated whether HSP60 could directly bind to the GTP using the NTP-agarose pull down assay. HSP60 could directly bind to the ATP-agarose (Fig. 1E). Similar to the result of the ATP-pull down assay, HSP60 could also bind directly to GTP (Fig. 1E). In these experiments, HSP10 did not bind to NTP-agarose. When the protein bound ATP- or GTP-agarose was eluted with each nucleotide, effective elution was detected for ATP-elution in contrast to GTP-elution (Fig. 1F). These results may be related to the fact that the turnover of GTPase activity was lower than that of ATPase. These results suggest that HSP60, but not HSP10, could directly bind to and hydrolyze both ATP and GTP.

### Difference in the interaction with co-chaperon HSP10 induced by the ATP- or GTPase activity

**Figure 2:**
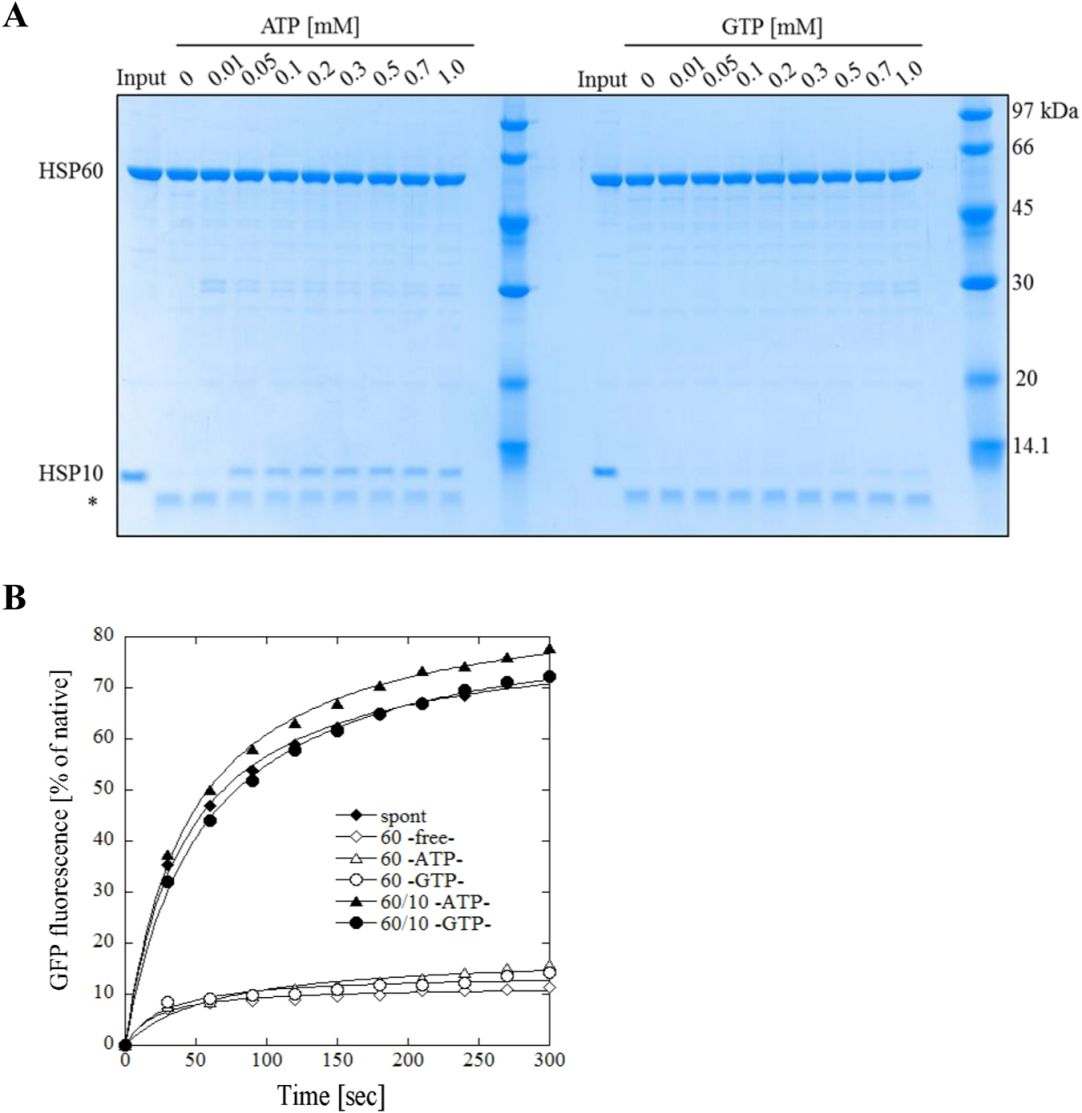
Interaction between HSP60 and HSP10 induced by ATP- or GTPase activity. (**A**), The interaction between HSP60 and HSP10 in the presence of ATP or GTP was evaluated by a trypsin sensitivity assay. Samples were separated by 12% SDS-PAGE and protein bands were detected by Coomassie Brilliant Blue R250-staining. Asterisk indicates the proteolytic fragment of HSP10. (**B**), Refolding assay of the acid-denatured GFP was performed to investigate the interaction between HSP60 and HSP10. The fluorescence recovery of GFP at 535 nm with excitation at 485 nm was measured for 300 sec.

We next investigated the interaction between HSP60 and HSP10 in two nucleotide-dependent manners. First, we performed a protease sensitivity assay. a HSP10 not bound to HSP60 is digested by trypsin, but HSP10 in complex to HSP60 is not digested^18^. ATP-dependent interaction between HSP60 and HSP10 was detected as full-length HSP10 protein bands in the ATP-concentration range from 0.05 to 1.0 mM, while GTP-dependent interaction was in the GTP-concentration range from 0.5 to 1.0 mM. The protein band signals with GTP were less than those of the ATP-dependent interaction (Fig. 2A and Figure S2A, B). To further investigate the interaction between HSP60 and HSP10, we next performed an acid-denatured GFP refolding assay. Refolding of acid-denatured GFP is dependent on spontaneous folding (Fig. 2B, closed diamond) and suppressed by HSP60 trapping the denatured GFP on the apical domain (Fig. 2B, closed blue diamond, open blue diamond, open triangle and open circle). Upon the addition of HSP10, the trapped denatured-GFP is released and effective refolding is initiated by binding HSP10 in the nucleotide-dependent reaction (Fig. 2B, closed triangle and closed circle). Taken together, these results suggest that HSP60 can interact with HSP10 in a GTPase-dependent manner, but either these interactions are weaker than those of the ATPase-dependent manner or the interval of the association and dissociation between HSP60 and HSP10 in the GTP-dependent manner is shorter than that of the ATP-dependent interaction; thus the GTPase activity of HSP60 is not suppressed.

### Nuccleotide-dependent oligomerization of HSP60 or HSP60/HSP10

**Figure 3:**
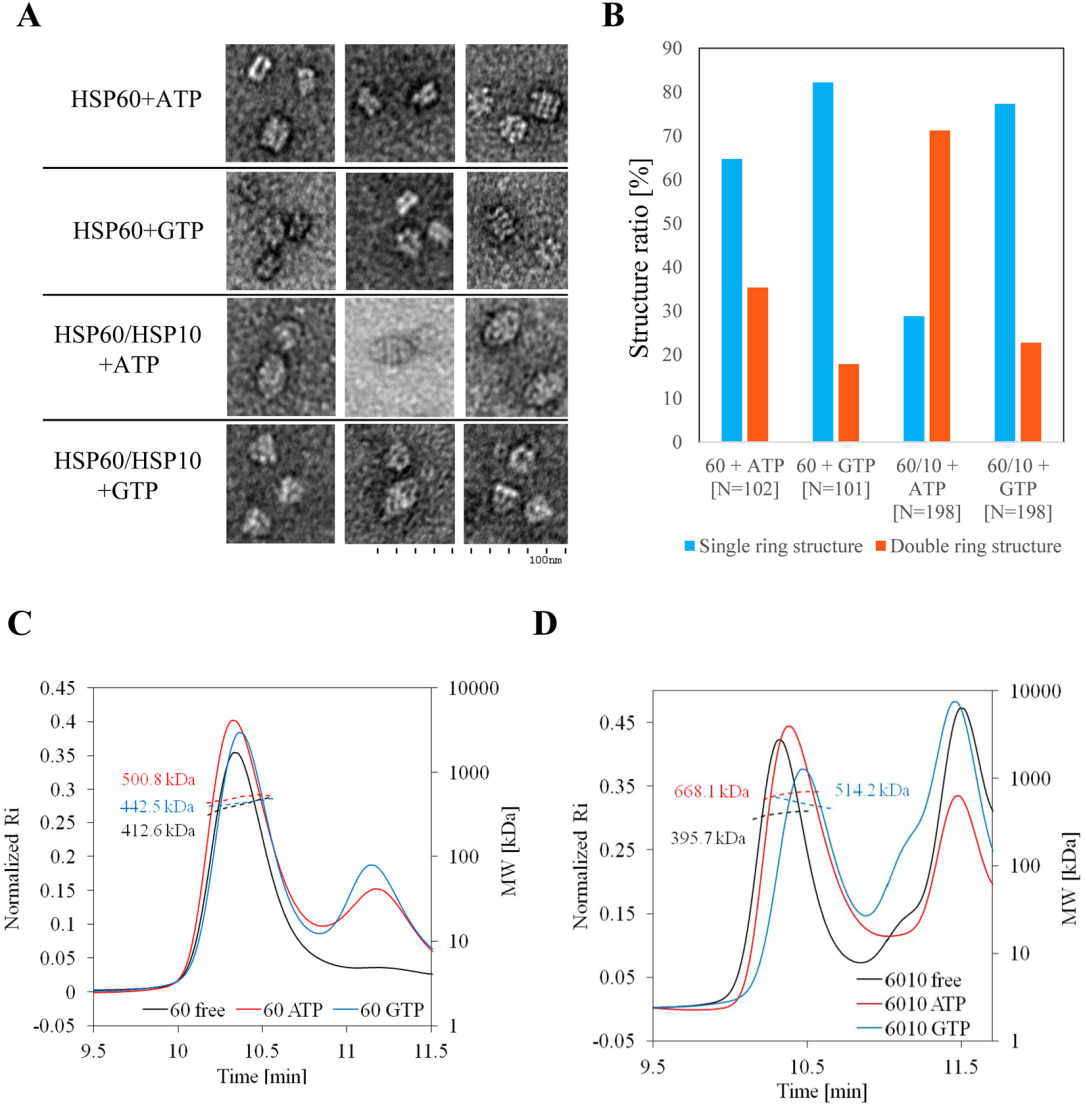
Oligomer-ization of HSP60 in the presence of ATP or GTP. (**A**), TEM images of HSP60 with or without HSP10 in the presence of ATP or GTP. Based on these images, statistical analysis was performed by counting 100 molecules (**B**). See also supplementary Figures S1A and S2C-E. **C** and **D**, SEC-MALS analysis of HSP60 with or without HSP10 in the presence of ATP or GTP. HSP60 with or without HSP10 was incubated for 5 min in the presence or absence of 1 mM nucleotides, then separated by gel filtration column chromatography. In the presence of nucleotides, the running buffer was treated with 1 mM ATP or GTP. The color of the detected Ri peak (solid line) and the molecular weight distribution (dashed line) corresponded. The molecular weight was calculated by Wyatt Astra Software.

We investigated the oligomerization of the HSP60 with or without HSP10 in the presence of ATP or GTP. We first used transmission electron microscopy and performed a statistical analysis of the side-view of HSP60 in the presence of ATP or GTP and presence or absence of HSP10 (Figures S2C to E). This analysis used over 100 molecules. As in the previous study^18^, the HSP60/HSP10 complex is classified as single-ring structures consisting of a single-ring (HSP60_7_) and single-ring complex (HSP60_7_-HSP10_7_), and the double-ring structures consisting of a double-ring (HSP60_14_), bullet-type complex (HSP60_14_-HSP10_7_) and football-type complex (HSP60_14_-(HSP10_7_)_2_), as shown in Figure S1B. In the presence of ATP, approximately 40% of the HSP60 became the double-ring (HSP60_14_). In agreement with a previous study^18^, approximately 70% of the HSP60 formed the double-ring structures mainly containing the football-type complex in the presence of both HSP10 and ATP (three panels on the first step and third step of Figures 3A,B and S2D). In the presence of GTP, approximately 20% of the HSP60 became the double-ring HSP60. Based on a comparison with ATP, the HSP60/HSP10 complex mainly formed single-ring structures mostly containing the single-ring complex (HSP60_7_-HSP10_7_) in the presence of GTP (three panels on the second step and fourth step of Figures 3A,B and S2E). These results suggest that the GTPase activity of HSP60 induces the interaction between HSP60 and HSP10, but hardly induces the the football-type complex compared with the ATPase activity.

To further investigate the oligomerization of the HSP60/HSP10 complex by the ATP- and GTPase activities, we performed a SEC-MALS analysis. This assay provides information about the stability of the HSP60/HSP10 oligomer^19^. In the absence of HSP10, an increase in the molecular weight of the HSP60 in comparison to the nucleotide-free condition was very slight, suggesting that the oligomerization of HSP60 was unstable in the presence of just ATP or GTP (Fig. 3C). It was reported by others that the oligomeric HSP60 was dissociated into the monomeric HSP60 in the presence of ATP^15^. Corresponding to this previous report, the minor peaks shown in Fig. 3C in the presence of ATP or GTP indicate the monomeric HSP60. In contrast, a clear increase in the molecular weight of HSP60 was displayed in the presence of both HSP10 and ATP, suggesting that the stability of the oligomeric state of HSP60 was increased by both HSP10 and ATP. In the presence of both HSP10 and GTP, the molecular weight of HSP60 increased, but the change was smaller than in the presence of both HSP10 and ATP (Fig. 3D). These results correspond with the TEM observations that the HSP60/HSP10 complex mainly formed the double-ring structures in the presence of ATP and the single-ring structures in the presence of the GTP. In Fig. 3D, the shoulder around 11 min indicated monomeric HSP60, and the large peaks around 11.5 min corresponded to HSP10. In the presence of ATP, the large peak corresponding to the HSP10 is clearly decreased in comparison with GTP, consistent with the strong interaction between HSP60 and HSP10 seen in the trypsin sensitivity assay (Fig. 2A). Taken together, these results suggest that the HSP60/HSP10 complex assumes the double-ring structures, containing mainly the football-type complex in the presence of ATP, and the single-ring structures, mainly containing the single-ring complex, in the presence of GTP.

### Folding properties of the GTPase-reaction cycle

**Figure 4:**
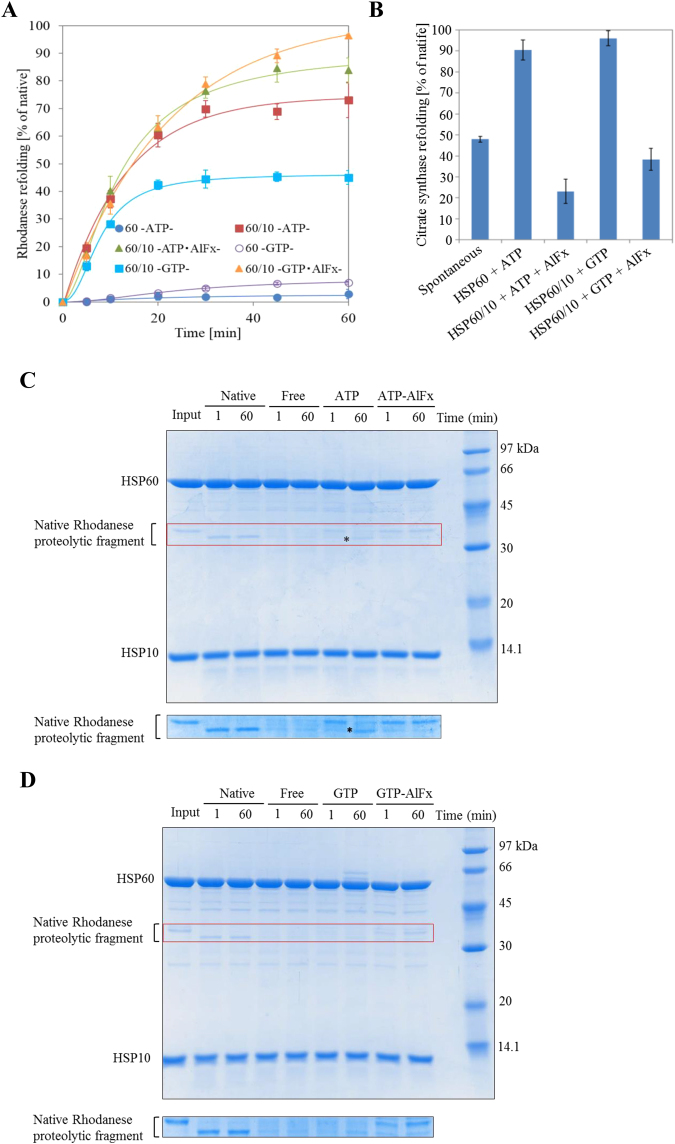
Folding properties of HSP60/HSP10 complex in the presence of ATP or GTP. (**A**), Refolding assay of the chemical-denatured rhodanese. The refolding yield of rhodanese was calculated by measuring the recovery of the enzymatic activity. The refolding reaction was performed for 60 min and the rhodanese activity was measured at the indicated times. (**B**), Refolding assay of the chemical-denatured citrate synthase. The refolding reaction was performed for 60 min and the refolding yield was calculated by measuring the recovery of the enzymatic activity. The in-cage folding of the HSP60/HSP10 complex was evaluated by a trypsin sensitivity assay using heat-denatured rhodanese in the presence or absence of ATP, ATP-AlFx (**C**), or GTP and GTP-AlFx (**D**). After initiation of the refolding reaction, samples were taken at 1 min or 60 min and treated with 10 μg/ml trypsin for 3 min. Samples were separated by 12% SDS-PAGE and the protein bands were detected by Coomassie Brilliant Blue R250-staining. The asterisk indicates the proteolytic fragment of the folded rhodanese digested by trypsin. The area of native rhodanese proteolytic fragments of the upper panels gels (the portion enclosed with a red frame) was analyzed under the high-resolution conditions (as shown in lower panels C and D). It is noted that heat denaturation has no effect on the function of HSP60 (see also supplementary Figure S3).

To investigate the physiological activities driven by the ATPase or GTPase, we performed the substrate refolding assay. We used the non-spontaneously refolding protein, rhodanese. The HSP60/HSP10 complex effectively refolded approximately 70% of the chemically denatured rhodanese in the presence of ATP (Fig. 4A). In the presence of GTP, the refolding yield was reduced to approximately 40% (Fig. 4A). In contrast, using the spontaneously refolding protein citrate synthase, the HSP60/HSP10 complex effectively refolds in the presence of both ATP and GTP (approximately 90%, Fig. 4B). This result agrees with the refolding of another spontaneous folding protein, GFP (Fig. 2B). We next performed the trypsin sensitivity assay to investigate the refolding of the heat-denatured rhodanese. The refolding yield of the heat-denatured rhodanese by HSP60/HSP10 is very similar to that of the chemically-denatured rhodanese, indicating that the function of HSP60 was not affected under the heat denaturation conditions. Denatured rhodanese trapped on the apical domain of HSP60 is completely digested by trypsin (Fig. 4C, lanes 4 and 5 from left). Native rhodanese, in contrast, is partially digested and produced a unique proteolytic fragment (Fig. 4C, lanes 2 and 3 from left). In the presence of ATP, denatured rhodanese, just after initiation of the folding reaction, was protected from trypsin digestion by encapsulation into the HSP60/HSP10 cavity (Fig. 4C, lane 6). Folded rhodanese was released from the cavity, digested by trypsin and produced the unique proteolytic fragment (Fig. 4C, asterisk in lane 7). However, in the presence of GTP, the denatured rhodanese was almost completely digested by trypsin for both reaction times, suggesting that the HSP60/HSP10 complex failed the effective encapsulation of the denatured rhodanese in the presence of GTP (Fig. 4D, lanes 6 and 7).

To investigate the influence of nucleotide hydrolysis, we used aluminum fluoride which mimics the hydrolysis-intermediate and limits the hydrolysis rate. With this treatment, the hydrolysis rates of ATP and GTP and the interaction between HSP60 and HSP10 became the same (Figures S2A and B). Moreover, the formation of the double-ring structures mainly containing the football-type complex was substantially increased (Figures S2F and G). Experiments with ATP-AlFx previously showed^16^ that the intrinsically-monomeric rhodanese was effectively encapsulated and refolded by the HSP60/HSP10 complex (Fig. 4A,C, Figure S3). However, with intrinsically-dimeric citrate synthase, the refolded monomeric citrate synthase was hardly released from the folding chamber, resulting in suppression of the refolding yield (Fig. 4B). The refolding yield of monomeric rhodanese by the GTPase activity of the HSP60/HSP10 complex was significantly increased by the treatment with aluminum fluoride (Fig. 4A and S3). In contrast, the refolding of the citrate synthase decreased, similar to ATP-AlFx (Fig. 4B). Moreover, the HSP60/HSP10 complex could protect the rhodanese from trypsin digestion by GTP-AlFx (Fig. 4D). Thus, the HSP60/HSP10 complex could mediate effective in-cage-folding by forcibly regulating the GTPase activity through the use of AlFx.

### ATPase-dependent activities of HSP60/HSP10 in the presence of GTP

**Figure 5:**
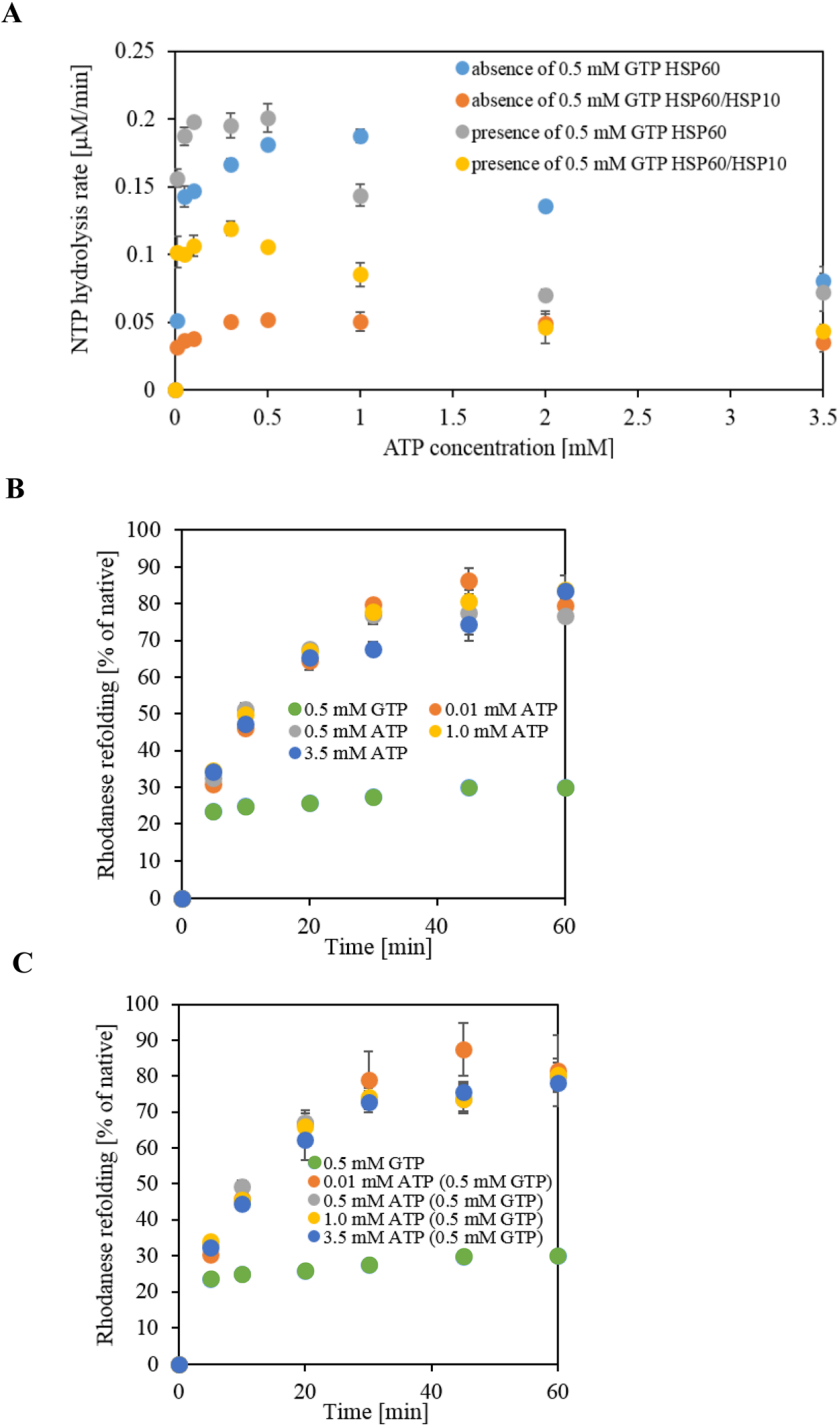
Enzymatic activity of HSP60 in the ATPase –dependent manner in the presence of GTP. (**A**), ATPase activity of HSP60 with or without HSP10 in the presence or absence of GTP. The NTP hydrolysis assay was performed in the presence of various concentrations of ATP with or without 0.5 mM GTP for 60 min. (**B**,**C**), Rhodanese refolding activity of HSP60/HSP10 in the presence of various concentrations of ATP with or without GTP. The refolding reaction was performed in the presence of 0.01, 0.5, 1.0 or 3.5 mM ATP with or without 0.5 mM GTP. The refolding yield was calculated by measuring the recovery of the enzymatic activity of rhodanese.

Since the cellular levels of GTP and ATP in the cytosol are estimated to be approximately 0.5 mM and 3.5 mM, respectively, we investigated the chaperone activity of HSP60/HSP10 in the presence of both ATP and GTP. We first examined the NTPase activity of HSP60 with or without HSP10 in the presence of 0.01 ~ 3.5 mM ATP and in the presence or absence of 0.5 mM GTP. The ATPase activity of HSP60 in the absence of GTP displayed a roll-off during the ATP hydrolysis at a concentration exceeding 1 mM (Fig. 5A, blue circles). This roll-off during ATP-hydrolysis has been reported for the bacterial group I chaperonin GroEL and displayed for the nested cooperativity model during the inter-ring negative cooperativity^20,25^. Upon the addition of GTP, the NTP hydrolysis rate of HSP60 was remarkably increased and the roll-off during the NTP hydrolysis was shifted at the lower ATP concentrations (exceeding 0.5 mM, Fig. 4A, gray circles). In the presence of HSP10, no significant roll-off in the ATP hydrolysis of HSP60 was detected (Fig. 5A, orange circle). However, upon the addition of GTP, the NTPase activity of HSP60 with HSP10 was remarkably increased and displayed a significant roll-off (exceeding 0.3 mM, Fig. 5A, yellow circles). Interestingly, the results of the NTPase activity of HSP60 or HSP60/HSP10 in the presence of 3.5 mM ATP with or without 0.5 mM GTP were almost all at the same levels. Therefore, it is postulated that the ATPase activity of HSP60 is not influenced by GTP at high concentrations of ATP, but with low concentrations of ATP, the activity may be influenced by the GTP.

We also investigated the ATP-dependent refolding activity of the HSP60/HSP10 (ATP concentrations 0.01, 0.5, 1.0 and 3.5 mM) with or without 0.5 mM GTP (Fig. 5B,C). The activity of HSP60/HSP10 in the presence of 0.5 or 1.0 mM GTP was characterized by low effective folding (approximately 30% of native, Fig. 5B,C). The refolding activity of the HSP60/HSP10 complex in presence or absence of 0.5 mM GTP displayed approximately 80% efficiency at all concentrations of ATP tested (Fig. 5B,C). As shown in Fig. 2A, significant interaction between HSP60 and HSP10 in the presence of ATP was not detected. However, since the ATPase activity of HSP60 in the presence of 0.01 mM ATP was suppressed by HSP10 (Fig. 1B), we postulated that HSP60 could bind to HSP10 and refold the non-native substrate. Considering these results in light of cellular nucleotide level conditions, it seems that the chaperone activity of the HSP60/HSP10 complex would not be influenced by GTP. Interestingly, at low ATP levels only the ATPase activity of the HSP60 or HSP60/HSP10 complex is affected by GTP.

### *In silico* 3D modeling of HSP60 bound by ATP or GTP

**Figure 6:**
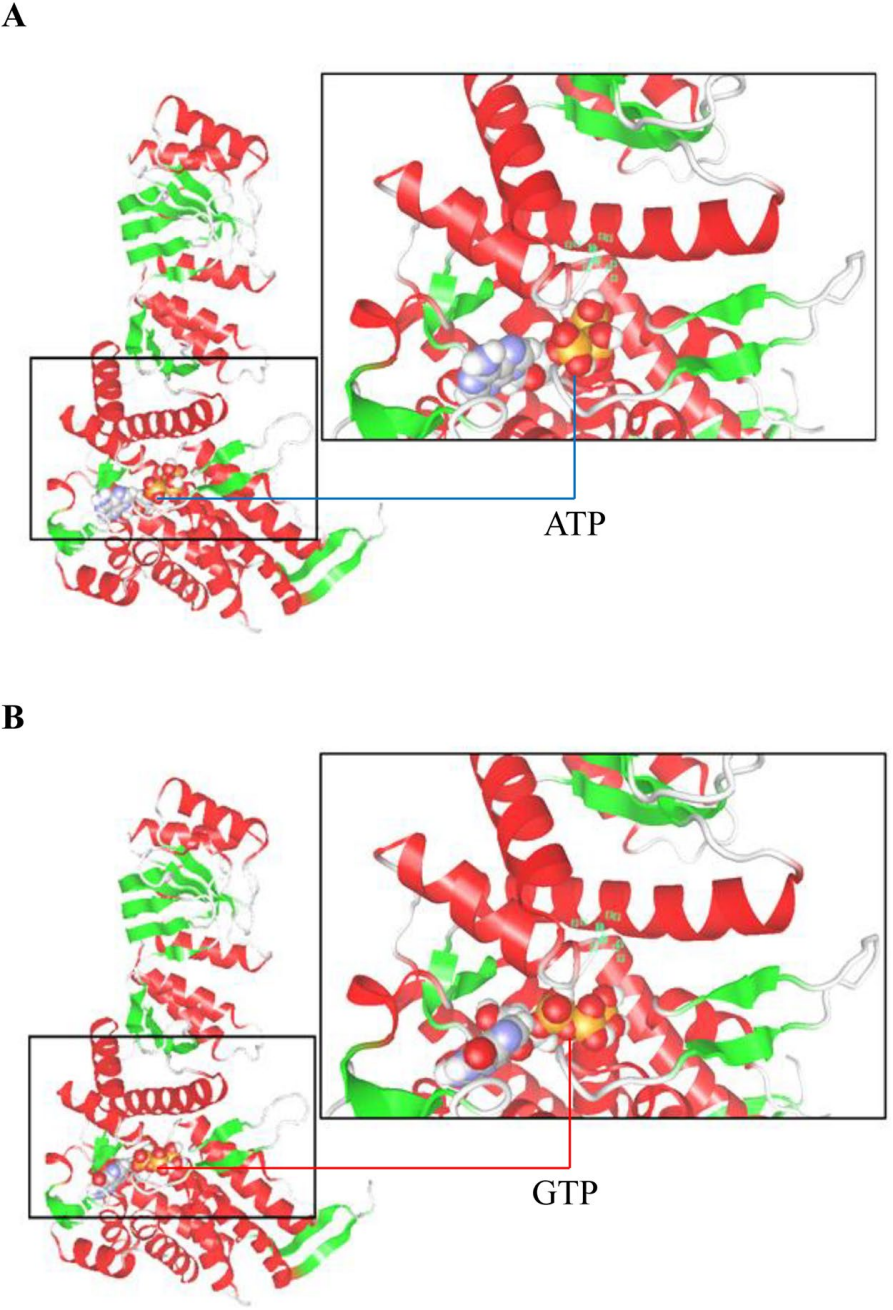
*In silico* modeling of the HSP60 binding ATP or GTP. (**A**) The 3D modeling of the HSP60 with ATP. (**B**) The 3D modeling of the HSP60 with GTP. The docking simulation was performed using MF myPresto v2 (FiatLux). The green boxes in the enlarged views showed the Asp398. The ribbon model shows the HSP60 (PDB entry is 4PJ1) and the ball models show the ATP and GTP (PDB entries are 1Q12 and 2YWQ, respectively).

To test whether GTP can bind to the nucleotide binding pocket of HSP60, we performed *in silico* docking simulation between HSP60 and NTPs. The result of the simulation with ATP is shown in Fig. 6A. Under the same conditions as Fig. 6A, GTP docked to the nucleotide binding pocket of HSP60 as well as ATP (Fig. 6B). In both Fig. 6A,B, the nucleotide binding pocket of HSP60 with ATP or GTP was magnified in the boxes. Asp398 plays a critical role in ATP hydrolysis. As shown in Fig. 6A,B, the distance between the γ-phosphate of GTP and Asp398 in these simulations was lengthened compared with the distance between ATP and Asp398. This decreased contact may explain the difference between the ATP and GTP hydrolysis. These results suggest that GTP may be able to bind to the nucleotide binding pocket of HSP60 as well as ATP.

## Discussion

It has been recently reported that the reaction cycle of the HSP60/HSP10 complex in the presence of ATP is distinct from that of the bacterial chaperonin GroEL/GroES^16,18,19^. In this study, we investigated the function and allostery of NTP hydrolysis activity at various concentrations of ATP and GTP. The ATPase activity of HSP60 was suppressed by HSP10, whereas GTPase activity unaffected. Moreover, the ATPase activities of HSP60 and HSP60/HSP10 displayed 2-step allosteric transitions in nucleotide concentration range from 0 to 1.0 mM, whereas the GTPase activity was only a single-step transition. Contrasting with results from TEM and SEC-MALS showing that the double-ring form of HSP60 was significantly increased in the presence of ATP, no significant increase in double-ring HSP60 was found in the presence of GTP. Thus, the two phases of ATP-dependent allosteric transitions of HSP60 correlate with an increase in double ring HSP60. In addition, since the interaction between HSP60 and HSP10 induced by the GTPase activity was weaker than that with ATP, these allosteric transitions might be involved in structural changes resulting in a state capable of binding HSP10. It is thought that this weak interaction affects the folding activity of the HSP60/HSP10 complex. Although HSP60/HSP10 could effectively refold an easily folding substrate like GFP and citrate synthase, HSP60/HSP10 hardly performed the effective folding of the non-spontaneous folding protein like rhodanese. Based on these results, HSP60 could bind to HSP10 in the presence of GTP alone and release the denatured substrate from the apical region, but the HSP60/HSP10 complex in the presence of GTP may fail to undergo encapsulation of the substrate.

In recent experiments with mutant proteins, it has been reported that the stability of the open state of HSP60 strengthens the interaction with HSP10 and stabilizes the formation of the double-ring structure^19,26^. Based on our results, because the interaction between HSP60 and HSP10 in the presence of GTP is weaker than with ATP, the encapsulated substrate may be released from the folding chamber before being completely folded. Moreover, the HSP60/HSP10 complex could not form the stable double-ring structures in the presence of GTP. These results suggest that formation of the stable double-ring structures is important for the effective in-cage folding during the HSP60/HSP10-reaction cycle.

At elevated ATP concentrations (from 1.0 to 3.5 mM), the ATPase activity of HSP60 was moderately reduced. This decrease has been already known from previous studies using GroEL^20^, and it is postulated that it suggests the nested allosteric model for the inter-ring negative cooperativity. In the presence of HSP10, the roll-off of the ATPase activity of HSP60 seemed to be reduced. A crystallographic study using the HSP60-HSP10 complex suggested that these complexes did not display significant inter-ring negative cooperativity. It seems that our results measuring the ATPase activity of HSP60 in the presence of HSP10 are consistent with previous reports. The inter-ring negative cooperativity of the HSP60 observed in this study occurred at higher concentrations of ATP than employed in a previous report using GroEL^20,25^, and these results also suggest that the inter-ring cooperativity of HSP60 was lower than that of GroEL.

The existence of the GTP affected the nucleotides hydrolysis curve. Particularly, the influence on the hydrolysis in the presence of HSP10 was high; the hydrolysis was promoted, and expressed a clear roll-off. In the presence of GTP, it was found that the roll-off in an ATP concentration dependent manner was initiated at a lower concentration in comparison to that in the absence of GTP. The experimental results applied the cytoplasmic nucleotide condition (3.5 mM ATP, 0.5 mM GTP) displayed the same hydrolysis levels in comparison with the absence of GTP. Even these suppressed the hydrolysis activity state, and the protein folding activity displayed a high efficiency. These results suggest that under cellular nucleotide concentrations (in this report as shown in Fig. 4, we applied the cytoplasmic nucleotide concentration), HSP60 may reduce consumption of the nucleotide and effectively mediate the protein folding.

In the current study, we investigated the ATPase and GTPase activities of mammalian wild type HSP60. These results describe a new insight into the active state of HSP60 under cellular nucleotide conditions. However, it does not become clear which nucleotides were preferentially used by HSP60 and what kind of influence it has on the single to double-ring transition or complex formation. It is suggested that solving these problems in the future can provide clues that reveal the functional mechanism of the HSP60-HSP10 complex.

## Materials and Methods

### Materials and proteins

ATP and GTP were purchased from Wako Pure Chemical Industries and purities of both reagents were 98%. ADP was purchased from Oriental Yeast Co. Ltd. GDP was purchased from Sigma. Porcine livers were purchased from Shibaura-zoki (Tokyo, Japan). The protocols for the animal experimentation described here were approved by the Animal Research Committee, Akita University. All subsequent animal experiments adhered to the “Guidelines for Animal Experimentation” of the university. The preparations of the mammalian wild-type HSP60 and HSP10 purified from porcine liver, His-tagged rhodanese and citrate synthase were previously described^18^. The GFP expression vector was kindly provided by Dr. Fumihiro Motojima (Toyama Prefectural University). The expression and purification of GFP was performed as already reported^21^. In all the experiments, the molar concentration of both HSP60 and HSP10 was calculated as a heptamer.

### Nucleotide hydrolysis assay

Measurement of the ATPase or GTPase activity was performed as previously reported^18^. The rate constant and Hill coefficient of the ATPase or GTPase activity of HSP60 (0.1 μM) or HSP60/HSP10 (0.1 μM/0.1 μM) were calculated by curve fitting to the Hill equation using KaleidaGraph (HULINKS). The ATPase activity was fitted to equation 1 ^24^.

1 $${V}_{{0}}=({V}_{max(1)}+{V}_{max(2)}{([S]/{K}_{{2}})}^{m})/(1+{({K}_{1}/[S])}^{{\rm{n}}}+{([S]/{K}_{2})}^{{\rm{m}}})$$

The ATPase activity at the low ATP concentration and GTPase activity were directly fitted to equation 2 ^20,23^.

2 $${V}_{{0}}={V}_{{\max }(1)}{K}_{{1}}{[S]}^{n}/(1+{K}_{{1}}{[S]}^{n})$$

In both equations, *V*_*0*_ is the initial NTPase velocity. The *V*_*max* (1)_ and *K*_1_ are the maximum NTPase velocity and equilibrium constant of the first allosteric transition, respectively, and *V*_max (2)_ and *K*_*2*_ are the maximum velocity and equilibrium constant of the second allosteric transition, respectively. For all the fitting curves in this article, the R^2^ values were greater than 0.99.

For the HPLC analysis, purified HSP60 (0.14 μM) was incubated with a nucleotide (final concentration of 50 μM) at 37 °C. The nucleotides were separated by C_18_ reverse phase column (SUPELCOSIL LC-18-T, 15 cm × 4.6 mm ID (3 µm particles) Shimadzu: Kyoto, Japan) chromatography with HPLC. The liquid chromatographic equipment consisted of a PU-1580 intelligent HPLC pump, LG-1580-02 ternary gradient unit (Jasco: Tokyo, Japan), SPD6A spectrophotometric detector, and CTO6A column oven (Shimadzu, Kyoto, Japan). Data were recorded and analyzed using a LabVIEW software system Version 7.1 (National Instruments: TX, USA). The chromatographic determination was performed at the flow rate of 1 ml/min at 37 °C, and the detection wavelength was set at 256 nm.

### Protease sensitivity assay

Aliquots of 1 μM HSP60 and HSP10 incubated in the presence of ATP or GTP indicated each concentration for 10 min at 25 °C. The aliquots were then treated with 10 μg/ml trypsin for 15 min at 25 °C. An encapsulation assay of the heat-denatured rhodanese by the trypsin treatment was previously performed^18^. The protease reaction was stopped by adding 1 mM PMSF and SDS loading buffer, then these samples were separated by 12% SDS-PAGE. The protein bands were detected by Coomassie Brilliant Blue R250-staining. The gels were analyzed by ChemiDoc MP Imaging System (BioRad, Hercules, California, USA).

*SEC-MALS analysis*. Aliquots of 1.23 μM HSP60 with or without 2.46 μM HSP10 were incubated in a column buffer (10 mM Tris-HCl pH 7.4, 10 mM MgCl_2_, 20 mM KCl) in the presence or absence of 1 mM ATP or GTP for 5 min at 25 °C. The aliquots were then separated by an SEC Protein Column for MALS (Wyatt Technology) equilibrated by the column buffer with or without 1 mM ATP or GTP at 25 °C. The column was connected to a DAWN HELEOS II 8 + (Wyatt technology) MALS detector and an Optilab T-rEX (Wyatt technology) reflective index/concentration detector. The obtained data were analyzed by Wyatt Astra Software (Wyatt Technology).

### Substrate protein refolding assay

The refolding assay of the chemically-denatured rhodanese and citrate synthase were performed as previously described^18^. The acid-denatured GFP solution (10 μM GFP, 0.1 M HCl, 50 mM HEPES-KOH pH 7.4) was diluted 200-fold into the assay buffer (50 mM HEPES-KOH pH 7.4, 10 mM MgCl_2_, 20 mM KCl, 1 mM DTT) containing 0.2 μM HSP60 and 0.01 mg/ml BSA. The GFP refolding was initiated by adding both ATP and HSP10 and the fluorescence intensity (485 nm of excitation and 535 nm of emission) monitored by a TECAN Infinite 200 (North Carolina, USA).

### NTP-pull down assay

ATP-agarose and GTP-agarose were purchased from Sigma-Aldrich. In this experiment, to strengthen the interaction between HSP60 and NTP-agarose, the aliquots were treated with aluminum fluoride. HSP60 (0.5 μM) or HSP10 (1.0 μM) was incubated with ATP- or GTP-agarose in the assay solution (50 mM HEPES-KOH pH7.4, 10 mM MgCl_2_, 20 mM KCl, 0.01% NP-40, 0.2 mM AlCl_3_, 10 mM NaF) for 60 min at 4 °C. The NTP-agarose after incubation was washed with the assay solution 5 times then boiled with the SDS-sample buffer in the presence of 10 mM EDTA and 1 M Urea at 100 °C for 5 min. In the case of elution, the NTP-agarose after washing was incubated with elution buffer (assay solution in the presence of 1 mM ATP or GTP) for 30 min at 4 °C. These samples were separated with 12% SDS-PAGE and detected by Coomassie Brilliant Blue R250-staining.

### *In silico* modeling of ATP or GTP binding HSP60

The docking simulation between HSP60 and NTP was performed by MF my Presto v2 (FiatLux, Tokyo, Japan). The 3D model of HSP60 used the PDB entry [4PJ1]. The 3D model of ATP and GTP used in these experiments was the PDB entry [1Q12] and [2YWQ], respectively.

## Electronic supplementary material

Supplementary Information
