# Supplementary material for: HSP60 possesses a GTPase activity and mediates protein folding with HSP10
Source: Sci Rep. 2017 Dec 5;7:16931. doi: 10.1038/s41598-017-17167-7 (PMC5717063; doi:10.1038/s41598-017-17167-7)
Supplement: Supplementary file 1 — Supplementary Information [file 41598_2017_17167_MOESM1_ESM.pdf]

Supplementary Information

HSP60 possesses a GTPase activity and mediates the protein folding with HSP10

Tomoya Okamoto[1], Hiroshi Yamamoto[1], Ikuru Kudo[1], Kazuya Matsumoto[2],
Masafumi Odaka[1], Grave Ewa[1], and Hideaki Itoh[1,*]

[1]*Department of Life Science, Graduate School and Faculty of Engineering Science, Akita University, Akita 010-8502, Japan*
[2]*Department of Applied Chemistry, Graduate School and Faculty of Engineering Science, Akita University, Akita 010-8502, Japan*

[*]To whom correspondence should be addressed: Hideaki Itoh, Department of Life Science, Graduate School and Faculty of Engineering Science, Akita University,
1-1 Tegata Gakuen Town, Akita University, Akita 010-8502, Japan.
Tel and Fax: +81-18-889-3041
E-mail: itohh@gipc.akita-u.ac.jp

Figure S1. Statistical analysis and NTPase activities of HSP60.

Statistical analysis of the HSP60 oligomer in the presence of ATP and GTP. **A**, TEM images of HSP60 in the absence of nucleotide. The side views show the single ring HSP60. Based on these images, statistical analysis was performed by counting 100 molecules (right graph). **B**, Purified HS P60 was incubated with ATP at 37 ºC for 2h. Sample of time 0 and 2 h were separated by a C_{18}-reverse phase column and absorbance at 256 nm was recorded. NTPase activity of GroEL and HSP10, and that of HSP60 in the presence of ADP or GDP. **C**, Nucleotide-hydrolysis assay was performed in the presence of 0.1 μM (tetradecamer) GroEL or HSP10. Free phosphate from nucleotide-hydrolysis was measured by the coloring reaction using Biomol green reagend (Enzo). **D**, The ATPase activity of HSP60 or HSP60/HSP10 in the presence or absence of ADP or GDP. NTP hydrolysis of HSP60 or HSP60/HSP10 was performed in the presence of 1 mM ATP with or without of 1 mM ADP or GDP for 60 min.

Figure S1

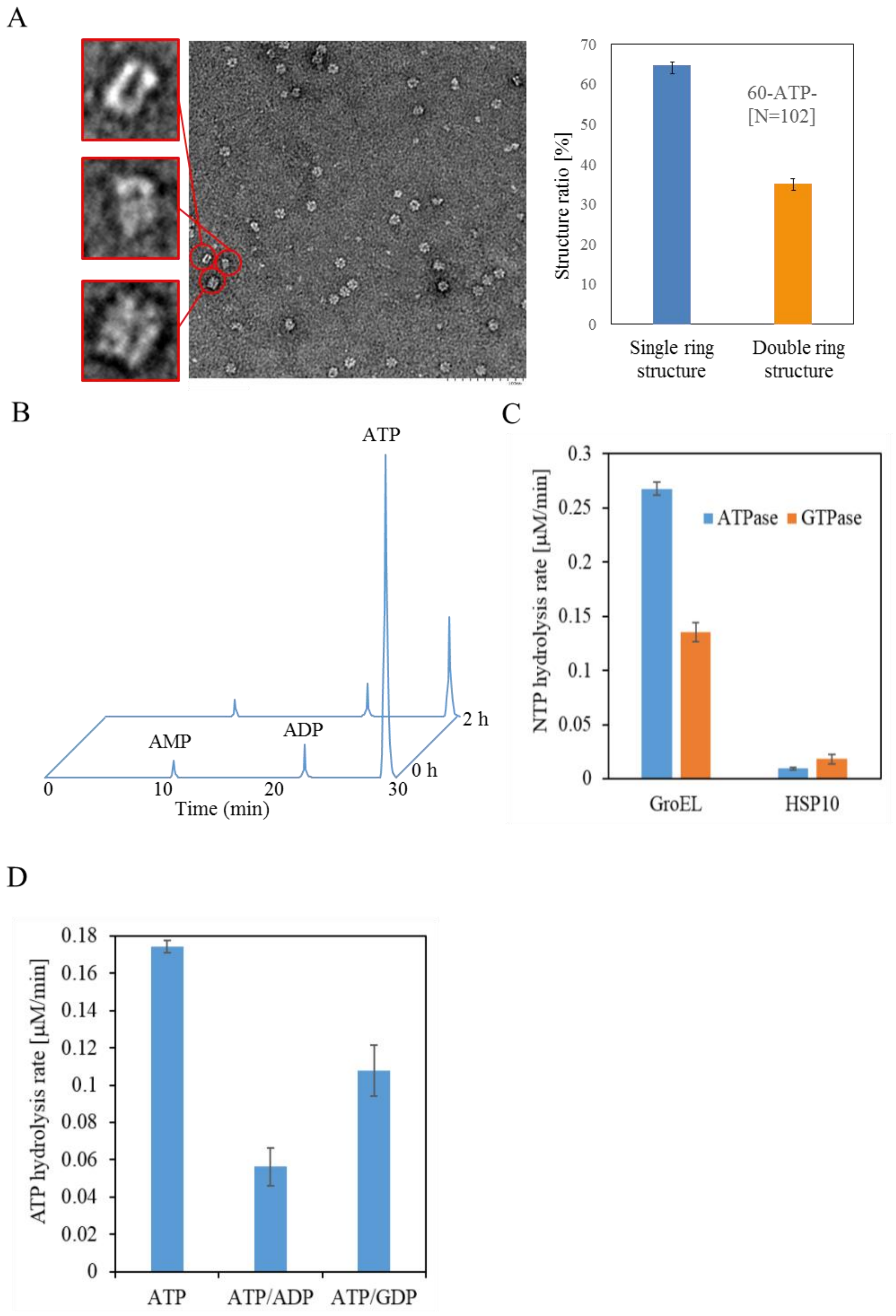
A
70
60
50
40
30
20
10
0
Structure ratio [%]
60-ATP-
[N=102]
Single ring structure
Double ring structure
B
ATP
AMP
ADP
2 h
0 h
0
10
20
30
Time (min)
C
0.3
0.25
0.2
0.15
0.1
0.05
0
NTP hydrolysis rate [μM/min]
ATPase
GTPase
GroEL
HSP10
D
0.18
0.16
0.14
0.12
0.1
0.08
0.06
0.04
0.02
0
ATP hydrolysis rate [μM/min]
ATP
ATP/ADP
ATP/GDP

Figure S2. Influence of ATP or GTP to structure and function of HSP60 and HSP10. Suppression of GTPae activity induces the strong interaction between HSP60 and HSP10. **A**, Nucleotide-hydrolysis assay of HSP60 was performed in the presence (green bar graph) or absence (blue bar graph) of 0.2 mM $AlCl_3$ and 10 mM NaF for 60 min at 25 ^{o}C. Nucleotide-hydrolysis was measured by the coloring reaction using Biomol Green reagent (Enzo). **B**, An interaction between HSP60 and HSP10 was examined by a trypsin sensitivity assay in the presence or absence of aluminum fluoride. Asterisk indicates the proteolytic fragment of the digested HSP10. Statistical analysis of the HSP60 oligomer in the presence of ATP or GTP. **C** to **E**, statistical analysis of the HSP60 or HSP60-HSP10 complex in the presence of nucleotide. **C**, Side views of HSP60 or HSP60/HSP10 complex were counted over 100 molecules from the TEM images under each condition. Side views obtained from TEM images are containing four oligomers and classified to the two structures. The single ring structures are consisted a single ring HSP60 ($HSP60_7$) and single ring complex ($HSP60_7$-$HSP10_7$), and the double ring structures are consisted a double ring HSP60 ($HSP60_{14}$), bullet-type complex ($HSP60_{14}$-$HSP10_7$) and football-type complex ($HSP60_{14}$-$(HSP10_7)_2$) as the main complex. N indicates total number of counted molecules of side views. **D**, Statistical analysis of HSP60 with or without HSP10 in the presence of ATP. **E**, Statistical analysis of HSP60 with or without HSP10 in the presence of GTP. Figures S1A and S2C- to E are related to Figure 3A and B. **F**, TEM analysis of HSP60/HSP10 complex in the presence of aluminum fluoride. Side view particles were counted for over 100 molecules and a statistical analysis performed (see also supplementary Figure S1-C). **G**, Statistical analysis of HSP60/HSP10 complex in the presence of ATP-AlFx or GTP-AlFx.

Figure S2

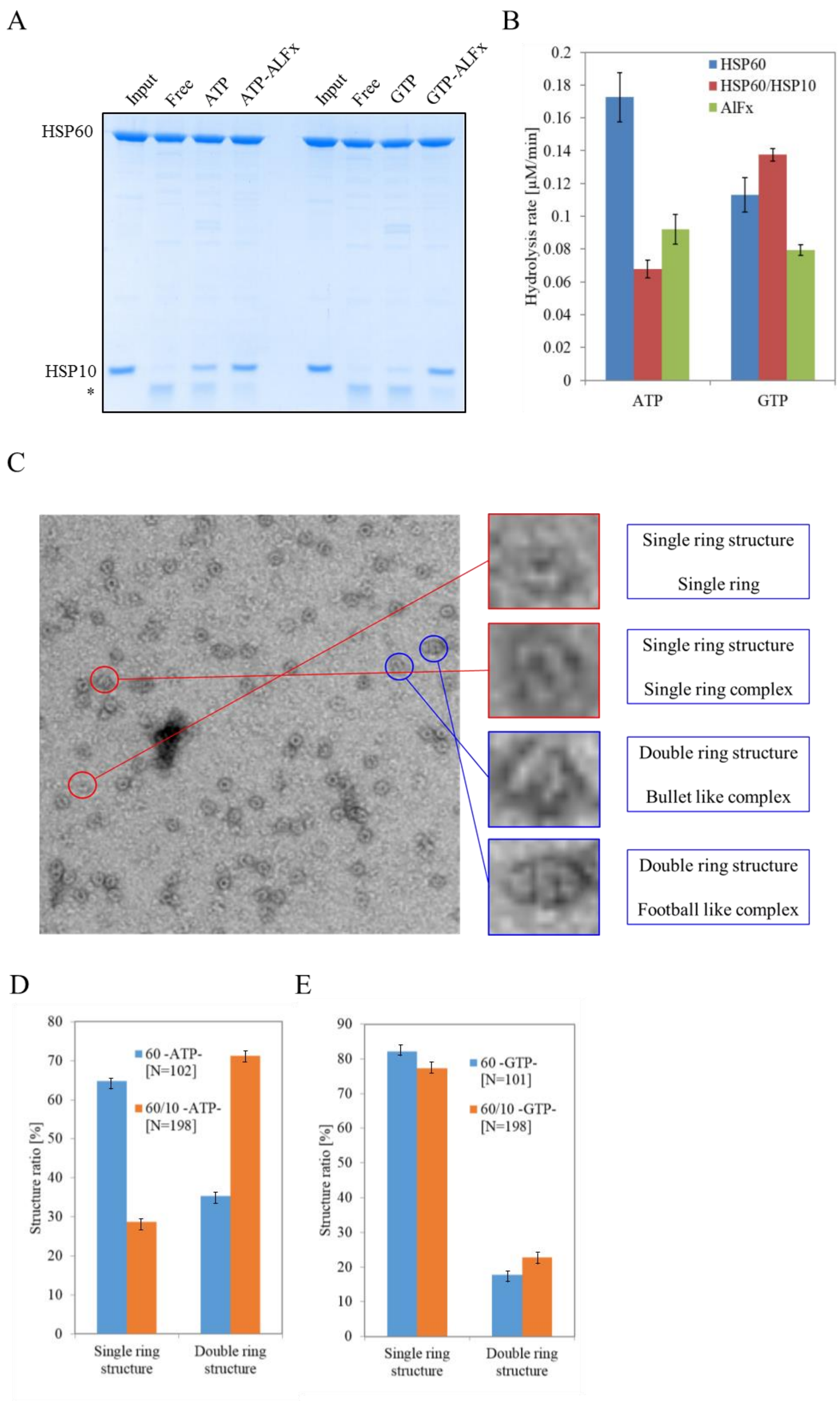
A
Input
Free
ATP
ATP-ALFx
Input
Free
GTP
GTP-ALFx
HSP60
HSP10
*
B
HSP60
HSP60/HSP10
AlFx
Hydrolysis rate [µM/min]
0.2
0.18
0.16
0.14
0.12
0.1
0.08
0.06
0.04
0.02
0
ATP
GTP
C
Single ring structure
Single ring
Single ring structure
Single ring complex
Double ring structure
Bullet like complex
Double ring structure
Football like complex
D
60 -ATP-
[N=102]
60/10 -ATP-
[N=198]
Structure ratio [%]
80
70
60
50
40
30
20
10
0
Single ring structure
Double ring structure
E
60 -GTP-
[N=101]
60/10 -GTP-
[N=198]
Structure ratio [%]
90
80
70
60
50
40
30
20
10
0
Single ring structure
Double ring structure

Figure S2

F

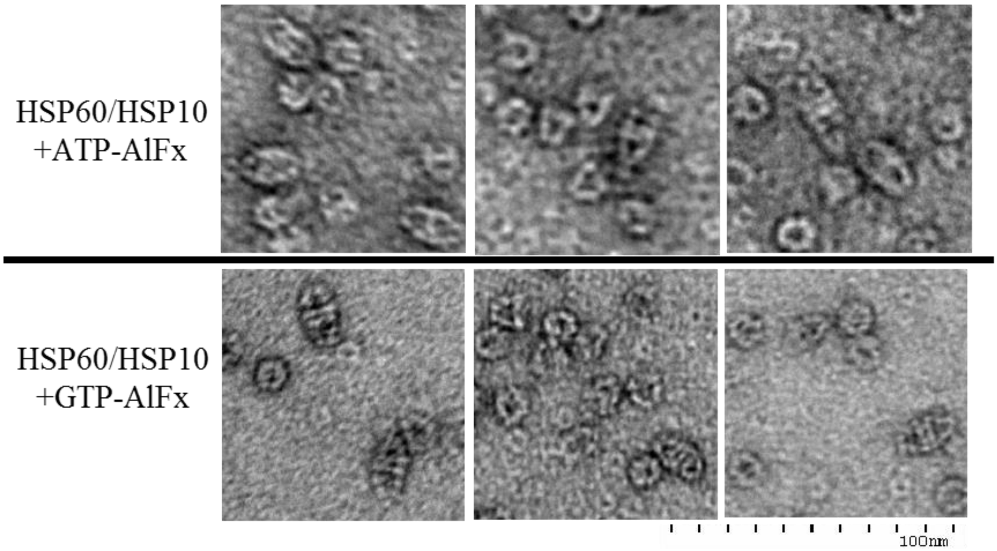
HSP60/HSP10
+ATP-AlFx
HSP60/HSP10
+GTP-AlFx
100nm

G

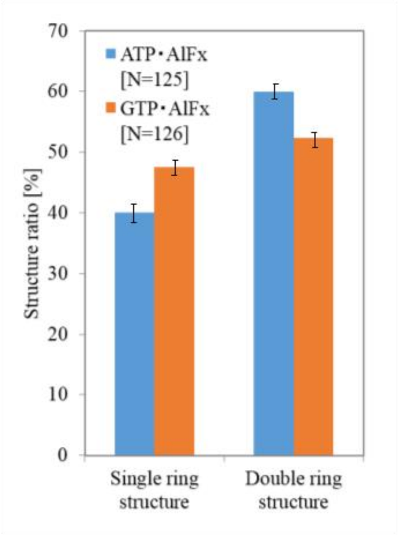
ATP・AlFx
[N=125]
GTP・AlFx
[N=126]
Structure ratio [%]
0
10
20
30
40
50
60
70
Single ring structure
Double ring structure

Figure S3. Refolding assay of the heat-denatured rhodanes.

A refolding assay of the heat-denatured rhodanese was performed to confirm that the heat denaturation had no effect to the HSP60 function. Rhodanese was denatured for 5 min at 55 ^{o}C in the presence of HSP60. After the denaturation, aliquots were preincubated for 10 min at 25 ^{o}C, then the refolding reaction was initiated by adding HSP10 and each nucleotide. The refolding reaction was performed for 60 min and the refolding yield was calculated by measuring the recovery of the rhodanese activity.

Figure S3

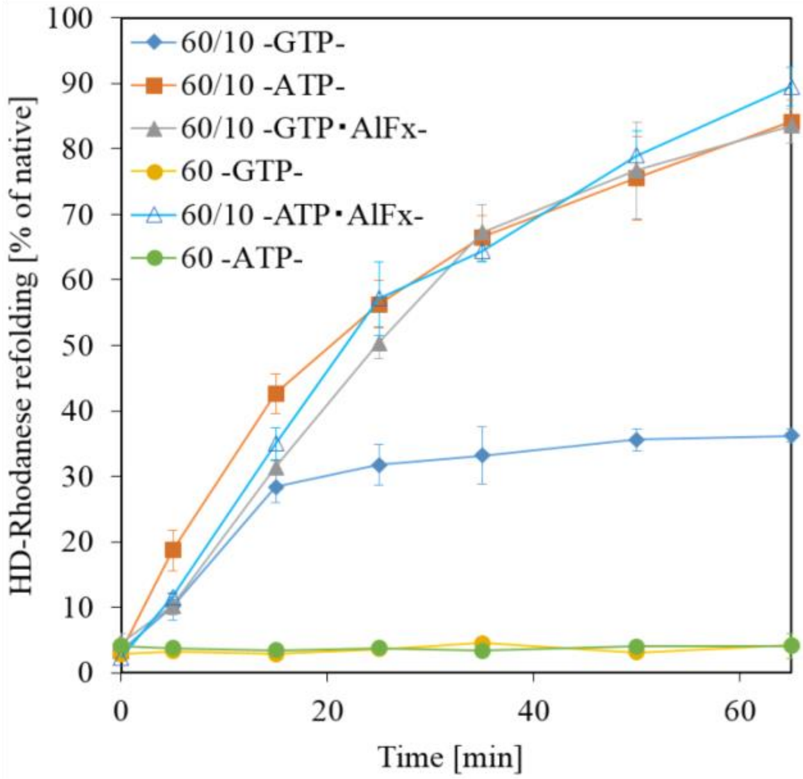

60/10 -GTP-
60/10 -ATP-
60/10 -GTP・AlFx-
60 -GTP-
60/10 -ATP・AlFx-
60 -ATP-
HD-Rhodanese refolding [% of native]
100
90
80
70
60
50
40
30
20
10
0
0
20
40
60
Time [min]
